# Mitochondrial dysfunction enhances cisplatin resistance in human gastric cancer cells via the ROS-activated GCN2-eIF2α-ATF4-xCT pathway

**DOI:** 10.18632/oncotarget.12356

**Published:** 2016-09-30

**Authors:** Sheng-Fan Wang, Meng-Shian Chen, Yueh-Ching Chou, Yune-Fang Ueng, Pen-Hui Yin, Tien-Shun Yeh, Hsin-Chen Lee

**Affiliations:** ^1^ Department and Institute of Pharmacology, School of Medicine, National Yang-Ming University, Taipei, Taiwan; ^2^ Department of Pharmacy, Taipei Veterans General Hospital, Taipei, Taiwan; ^3^ School of Pharmacy, Taipei Medical University, Taipei, Taiwan; ^4^ National Research Institute of Chinese Medicine, Ministry of Health and Welfare, Taipei, Taiwan; ^5^ Institute of Medical Sciences, Taipei Medical University, Taipei, Taiwan; ^6^ Department of Medical Research, Taipei Veterans General Hospital, Taipei, Taiwan; ^7^ Institute of Anatomy and Cell Biology, School of Medicine, National Yang-Ming University, Taipei, Taiwan

**Keywords:** gastric cancer, mitochondrial dysfunction, cisplatin resistance, xCT, retrograde signaling

## Abstract

Mitochondrial DNA mutations and defects in mitochondrial enzymes have been identified in gastric cancers, and they might contribute to cancer progression. In previous studies, mitochondrial dysfunction was induced by oligomycin-enhanced chemoresistance to cisplatin. Herein, we dissected the regulatory mechanism for mitochondrial dysfunction-enhanced cisplatin resistance in human gastric cancer cells. Repeated cisplatin treatment-induced cisplatin-resistant cells exhibited high SLC7A11 (xCT) expression, and xCT inhibitors (sulfasalazine or erastin), xCT siRNA, or a GSH synthesis inhibitor (buthionine sulphoximine, BSO) could sensitize these cells to cisplatin. Clinically, the high expression of xCT was associated with a poorer prognosis for gastric cancer patients under adjuvant chemotherapy. Moreover, we found that mitochondrial dysfunction enhanced cisplatin resistance and up-regulated xCT expression, as well as intracellular glutathione (GSH). The xCT inhibitors, siRNA against xCT or BSO decreased mitochondrial dysfunction-enhanced cisplatin resistance. We further demonstrated that the upregulation of the eIF2α-ATF4 pathway contributed to mitochondrial dysfunction-induced xCT expression, and activated eIF2α kinase GCN2, but not PERK, stimulated the eIF2α-ATF4-xCT pathway in response to mitochondrial dysfunction-increased reactive oxygen species (ROS) levels. In conclusion, our results suggested that the ROS-activated GCN2-eIF2α-ATF4-xCT pathway might contribute to mitochondrial dysfunction-enhanced cisplatin resistance and could be a potential target for gastric cancer therapy.

## INTRODUCTION

Gastric cancer is one of the most common cancers worldwide. The incidence rate of gastric cancer was highest in East Asia, Eastern Europe, and South America [1]. The main treatment modality for gastric cancer is surgery, except for in the advanced or unresectable stages. Systemic chemotherapy based on 5-fluorouracil (5-FU) plus an anthracycline agent or cisplatin-containing combinations, is the most effective treatment modality for patients with metastatic disease [2]. However, chemotherapy resistance remains an important issue in the treatment of advanced gastric cancer patients.

Cancer cells usually exhibit an aberrant metabolism, and deregulated cellular energetics were recently included among the hallmarks of cancer [3]. Mitochondria are intracellular organelles that participate in bioenergetics metabolism and cellular homeostasis, including the generation of adenosine triphosphate (ATP) through oxidative phosphorylation, intermediate metabolism, the production of reactive oxygen species (ROS), and the regulation of apoptosis [4]. In past decades, somatic mutations in the mitochondrial genome have been found to be frequent events in human gastric cancers, and most of these mitochondrial DNA (mtDNA) mutations can lead to mitochondrial dysfunction [5, 6]. Mitochondrial dysfunction has been suggested to contribute to cancer progression [7, 8].

Accumulating evidence has revealed that the mitochondria can generate mitochondria-to-nucleus (retrograde) signals to regulate cellular function and to protect against mitochondrial dysfunction by activating the expression of nuclear genes involved in metabolic reprogramming or stress defense [7]. The retrograde signaling pathways have thus been proposed to be involved in mitochondrial dysfunction-enhanced malignant progression [8]. In our previous study, mitochondrial dysfunction was found to promote a migration phenotype and cisplatin resistance in human gastric cancer cells [6]. Recently, various retrograde signaling pathways have been shown to contribute to mitochondrial dysfunction-enhanced cancer migratory/invasive abilities [9, 10]. However, the molecular mechanism underlying mitochondrial dysfunction-induced cisplatin resistance remains unclear.

Several mechanisms can result in cisplatin resistance, including reduced intracellular drug accumulation, increased inactivation by thiol-containing molecules, e.g. glutathione (GSH), increased DNA damage repair, and the inhibition of apoptosis [11]. Among these mechanisms, increases in GSH have been demonstrated in a number of cisplatin-resistant tumor models and have been confirmed in clinical studies [11]. Moreover, elevated GSH might increase DNA repair or increase the inhibitory effect on apoptosis by buffering drug-induced oxidative stress [12]. An increased intracellular GSH level is thus generally accepted to be a significant mechanism for cisplatin resistance.

The system x_c_^−^ cystine/glutamate antiporter is a plasma membrane transporter mediating the cellular uptake of cystine from the microenvironment in exchange for intracellular glutamate [13]. This transporter is known to contribute to the maintenance of intracellular GSH levels and to protect cells from oxidative stress or toxic compounds [13]. Human system x_c_^−^ cystine/glutamate antiporter consists of the heavy subunit 4F2hc (found in a variety of amino-acid transporters) and the light subunit xCT (SLC7A11). The transport activity is thus believed to be determined by xCT [13]. It was reported that xCT gene expression could be up-regulated by nuclear factor erythroid 2-related factor-2 (Nrf2), which binds to antioxidant response elements (AREs) [14], or by the activating transcription factor 4 (ATF4), which binds to amino acid response elements (AAREs) in the xCT promoter [15, 16].

The eukaryotic initiation factor 2α (eIF2α)-ATF4 pathway is the central regulator of the integrated stress response. The phosphorylation of Ser-51 of the eukaryotic initiation factor eIF2α attenuates the initiation of global cap-dependent protein translation, but it concurrently increases the cap-independent translation of select mRNAs with small upstream open reading frames, including the ATF4 transcription factor [17]. The increase in eIF2α phosphorylation leads to the adaptation of the cell to stress conditions or, alternatively, to the induction of apoptosis [17, 18]. Moreover, four eIF2α kinases have been identified in mammals, and dsRNA-activated protein kinase R (PKR), heme-regulated inhibitor eIF2α kinase (HRI), protein kinase R-like endoplasmic reticulum kinase (PERK), and general control nonderepressible-2 (GCN2) are activated by distinct stresses [17]. Recent reports have suggested that GCN2 or PERK might contribute to increased eIF2α phosphorylation during different types of mitochondrial stress [19, 20]. The activation of the eIF2α-ATF4 pathway and the eIF2α kinases might be involved in retrograde signaling pathways.

In this study, we evaluated the role of xCT in the mitochondrial dysfunction-enhanced cisplatin resistance of human gastric cancer cells. Cisplatin-resistant gastric cancer cells were established to validate high xCT expression in cisplatin resistance. Moreover, the molecular mechanisms involved in the eIF2α-ATF4 pathway and the eIF2α kinases were investigated.

## RESULTS

### Cisplatin-resistant gastric cancer cells have high xCT expression and rely on environmental cystine for cell survival

**Figure 1:**
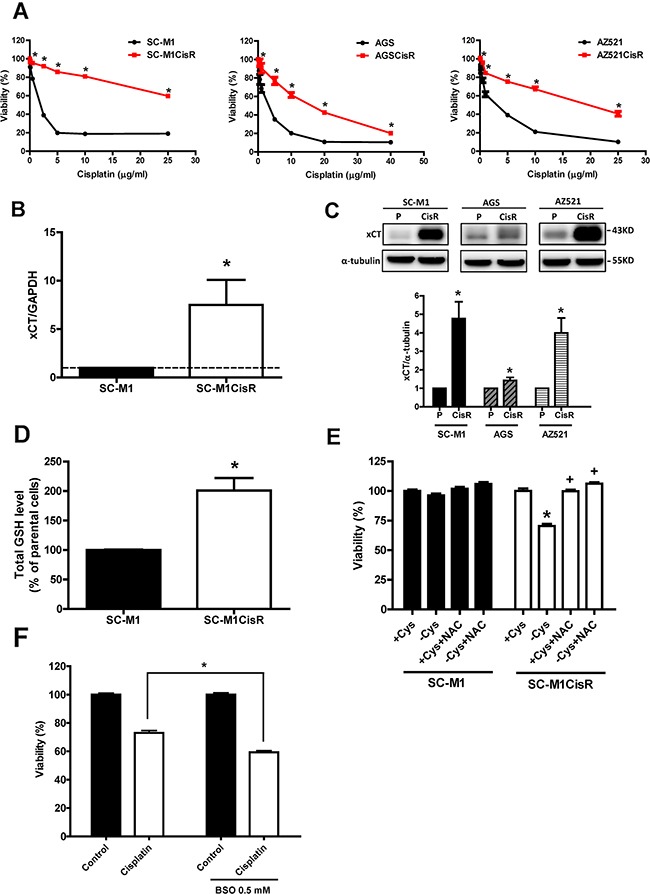
Cisplatin-resistant gastric cancer cells have high xCT expression and rely on environmental cystine for cell survival **A.** The cisplatin-resistant gastric cancer cells (cisplatin-resistant, CisR) and their parental SC-M1, AGS, and AZ521 cells were treated with cisplatin for 48 h. The sensitivity was determined by SRB assay. **B.** qRT-PCR analysis of xCT mRNA between the SC-M1 and the cisplatin-resistant SC-M1 gastric cancer cells. qRT-PCR values were normalized to GAPDH mRNA. **C.** Western blot analysis of xCT protein expression between the parental and the cisplatin-resistant gastric cancer cells. **D.** The parental SC-M1 and cisplatin-resistant SC-M1 cells (SC-M1CisR) were seeded at a density of 1 × 10^6^ cells per 10-cm dish. After culture overnight, the cells were collected and the total cellular GSH levels were analyzed by GSH assay kit. **E.** The SC-M1 parental and cisplatin-resistant (SC-M1CisR) gastric cancer cells were seeded at a density of 3000 cells per well in 96-well plates and were cultured in cystine-free medium (−Cys) and cystine-containing complete medium (+Cys). The cells were pre-treated with N-acetyl cysteine (NAC, 5 mM) for 12 h, followed by co-treatment with cystine-free medium and cystine-containing complete medium for 72 h. **F.** The SC-M1CisR cells were treated with BSO and/or cisplatin (25 μg/ml) for 48 h. The cell viability was determined by SRB assay. Data represent the mean ± SEM of three independent experiments. *p < 0.05, compared to the parental cells or + Cys group; + *p* < 0.05, compared to the -Cys group.

To establish cisplatin-resistant gastric cancer cells, we repeatedly treated gastric cancer cells with increasing concentrations of cisplatin, starting from a low dose. We obtained three lines of cisplatin-resistant gastric cancer cells derived from SC-M1, AGS, and AZ521 cells. Figure 1A shows that these cisplatin-resistant gastric cancer cells exhibited a significantly decreased sensitivity to cisplatin (IC50 of cisplatin: 2.23 μg/ml for SC-M1, >25 μg/ml for SC-M1CisR; 2.31 μg/ml for AGS, 15.83 μg/ml for AGSCisR; 2.39 μg/ml for AZ521, 18.71 μg/ml for AZ521CisR). We found that the protein expression of multidrug-resistance protein (MDR) and the function of MDR1 (p-glycoprotein, P-gp) and multidrug resistance-associated protein (MRP) were not increased in the cisplatin-resistant cells, compared to the parental cells (Supplementary Figures S1A-S1C). Moreover, these cisplatin-resistant cancer cells had higher xCT gene and protein expression (Figures 1B and 1C). In addition, higher intracellular GSH levels (Figure 1D) were observed, compared with their parental SC-M1 cells. We also found that the cisplatin-resistant cells were more sensitive to cystine-depletion medium than the parental cells, and N-acetyl cysteine (NAC) reversed cystine depletion-induced cell death (Figure 1E). Moreover, buthionine sulphoximine (BSO, a GSH biosynthesis inhibitor) treatments increased the sensitivity of the cisplatin-resistant cells to cisplatin (Figure 1F). These results suggested that xCT-dependent GSH elevation might play an important role in the cell survival of cisplatin-resistant gastric cancer cells. Moreover, the basal ROS level in the cisplatin-resistant gastric cancer cells was lower than in the parental cells (Supplementary Figures S1D and S1E). Cisplatin resistance and cell survival might be ROS-dependent in cisplatin-resistant gastric cancer cells.

### Inhibition and knockdown of xCT increase the cisplatin sensitivity of the cisplatin-resistant gastric cancer cells, and high xCT expression is a poor prognostic factor in gastric cancer patients under adjuvant chemotherapy treatment

**Figure 2:**
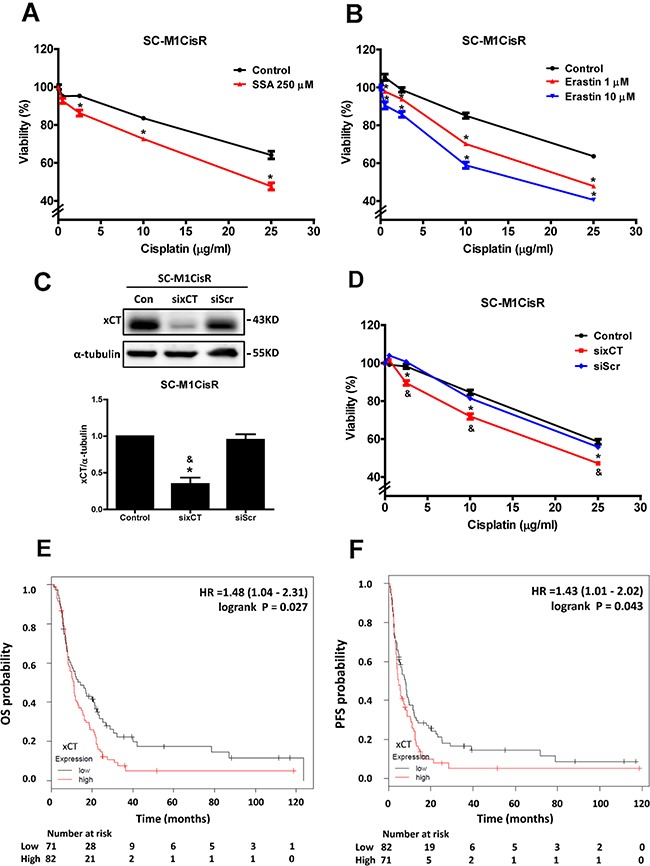
Inhibition and knockdown of xCT increase the cisplatin sensitivity of cisplatin-resistant gastric cancer cells, and high xCT expression is a poor prognostic factor in gastric cancer patients under adjuvant chemotherapy treatment **A.** The SC-M1CisR cells were treated with cisplatin and SSA for 48 h. The cell viability was determined by MTT assay. **B.** The SC-M1CisR cells were treated with cisplatin and erastin for 48h. The cell viability was determined by SRB assay. **C.** Specific siRNA against xCT (60 pmol for 4 × 10^5^ cells in a 6-cm dish) was used to knock down xCT in the SC-M1CisR cells, and the protein level of xCT was analyzed by Western blot analysis. (siRNA for non-target sequence, siScramble, siScr) **D.** The xCT-silenced SC-M1CisR cells (sixCT) and the control SC-M1CisR cells were treated with cisplatin for 48 h. The cell viability was determined by SRB assay. (E, F) The Kaplan-Meier survival analyses show the effects of xCT expression on overall survival (OS) **E.** and progression-free survival (PFS) **F.** in the subgroup of gastric cancer patients (5-FU-based adjuvant gastric cancer patients). Data represent the mean ± SEM of three independent experiments. *p < 0.05, compared to the control group; & *p* < 0.05, compared to the individual siScr group.

To evaluate further whether high xCT expression is essential for cisplatin resistance, we treated the cisplatin-resistant cells with xCT inhibitors, such as sulfasalazine (SSA) and erastin. The results revealed that SSA and erastin partially increased the cell's sensitivity to cisplatin (Figures 2A and 2B). In addition, the knockdown of xCT expression by siRNA increased the cell's cisplatin sensitivity (Figures 2C and 2D). These results suggested that increased xCT expression could contribute to cisplatin resistance in human gastric cancer cells.

To understand the clinical impacts of xCT expression in gastric cancer patients, we used an online-database (http://kmplot.com/analysis/) and analyzed the effect of xCT expression on overall survival (OS) and progression-free survival (PFS) in gastric cancer patients with chemotherapy treatment. In gastric cancer patients within the clinical cohort undergoing adjuvant chemotherapy (n=153), we found that high xCT-expressing gastric cancer patients had a lower OS (hazard ratio [HR]: 1.48, 1.04-2.31, log rank *p* = 0.027, Figure 2E) and a lower PFS (HR: 1.43, 1.01-2.02, log rank *p*= 0.043, Figure 2F) than low xCT-expressing patients. These results suggested that high xCT expression is a poor prognostic factor for gastric cancer patients receiving adjuvant chemotherapy treatment.

### Mitochondrial dysfunction enhances cisplatin resistance and increases xCT expression in human gastric cancer cells

**Figure 3:**
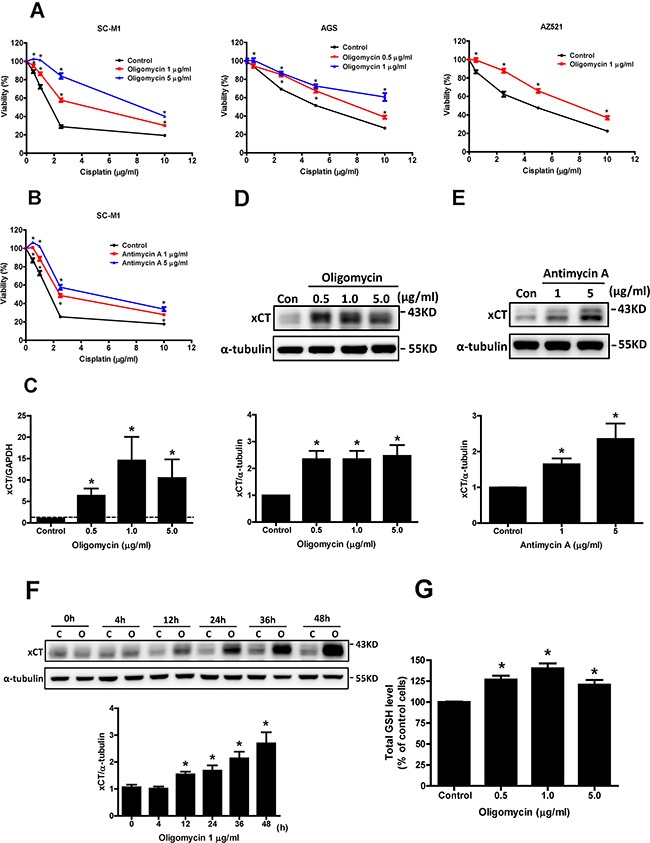
Mitochondrial dysfunction enhances cisplatin resistance and increases xCT expression in human gastric cancer cells **A.** Human gastric cancer cells SC-M1, AGS, and AZ521 were treated with cisplatin and oligomycin for 48 h. **B.** The SC-M1 cells were treated with cisplatin and antimycin A for 48 h. The cell viability was determined by SRB assay. **C.** qRT-PCR analysis of xCT mRNA in the SC-M1 cells under oligomycin treatment for 24 h. qRT-PCR values were normalized to GAPDH mRNA. (D, E) Western blot analysis of xCT protein expression in the SC-M1 cells under oligomycin **D.** and antimycin A **E.** treatments for 24 h, respectively. **F.** The SC-M1 cells were treated with oligomycin for 0-48 h (C: control; O: oligomycin). The changes of xCT protein expression were analyzed by Western blot. **G.** For glutathione (GSH) assay, the SC-M1 cells were seeded at a density of 1 × 10^6^ cells per 10-cm dish. After oligomycin treatments for 24 h, the cells were collected, and the total cellular GSH level was analyzed with a GSH assay kit. Data represent the mean ± SEM of three independent experiments. *p < 0.05, compared to the control group.

Three human gastric cancer cell lines (SC-M1, AGS, and AZ521) were treated with 0.5-5 μg/ml oligomycin (a Complex V ATPase inhibitor) or 1-5 μg/ml antimycin A (a Complex III inhibitor) to induce mitochondrial dysfunction, as in our previous studies [6, 9, 21, 22]. We found that sensitivity to cisplatin was decreased in the cells with oligomycin co-treatment, compared to those without oligomycin treatment (Figure 3A). The decrease in sensitivity to cisplatin was also observed in the gastric cancer cells treated with antimycin A (Figure 3B). In addition, we found that both oligomycin- and antimycin A-induced mitochondrial dysfunction could increase the gene expression of xCT (Figure 3C), and its protein expression (Figures 3D and 3E). The up-regulation of xCT expression by oligomycin treatment occurred in a time-dependent manner (Figure 3F). Based on the function of the system x_c_^−^ transporter, we determined intracellular GSH levels and found that intracellular GSH was increased by oligomycin-induced mitochondrial dysfunction (Figure 3G). These results suggested that mitochondrial dysfunction could enhance cisplatin resistance and increase xCT expression in human gastric cancer cells.

### Inhibition and knockdown of xCT reduce mitochondrial dysfunction-enhanced cisplatin resistance

**Figure 4:**
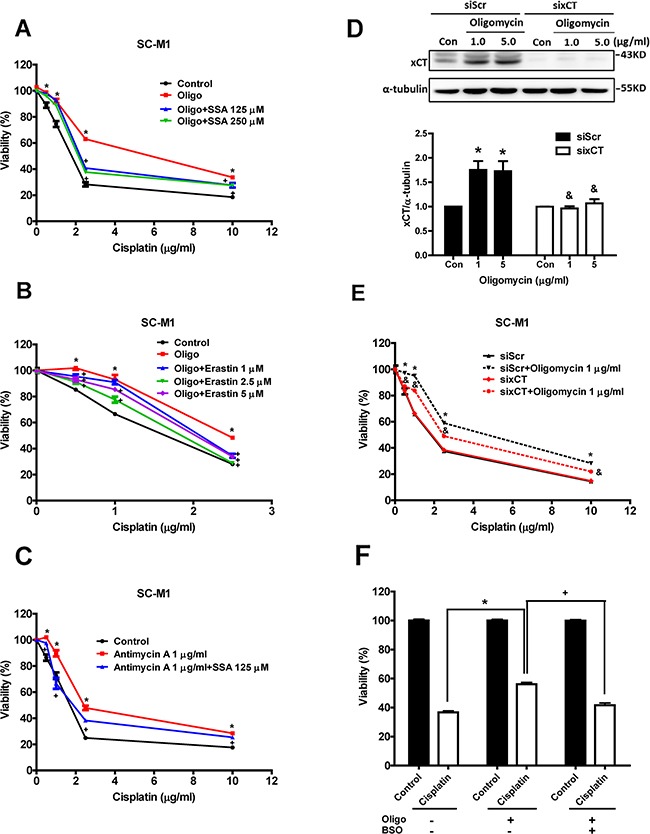
Inhibition and knockdown of xCT reduce mitochondrial dysfunction-enhanced cisplatin resistance (A, B) The SC-M1 cells were treated with oligomycin, cisplatin, and sulfasalazine (SSA) **A.** or erastin **B.** for 48 h. (Oligo: oligomycin 1 μg/ml). **C.** The SC-M1 cells were treated with antimycin A, cisplatin, and SSA for 48 h. The cell viability was determined by SRB assay. **D.** Western blot analysis of the xCT protein expression in the xCT-silenced SC-M1 cells (sixCT) and the control cells (siRNA for non-target sequence, siScramble, siScr) under oligomycin treatment for 24h. **E.** Specific siRNA (60 pmol for 4 × 10^5^ cells in a 6-cm dish) against xCT was used to knock down xCT in the SC-M1 cells. The cells were treated with oligomycin and cisplatin for 48 h. **F.** The SC-M1 cells were treated with oligomycin, cisplatin (2.5 μg/ml) and/or buthionine sulphoximine (BSO, 0.5 mM) for 48 h. The cell viability was determined by SRB assay. Data represent the mean ± SEM of three independent experiments. *p < 0.05, compared to the control group; + *p* < 0.05, compared to the oligomycin alone group; & *p* < 0.05, compared to the individual siScr group.

To evaluate whether the increased xCT expression contributed to cisplatin resistance, we used two xCT inhibitors (SSA and erastin) to inhibit xCT function. We found that both SSA and erastin could significantly reduce oligomycin-induced cisplatin resistance (Figures 4A and 4B). Antimycin A-induced cisplatin resistance was also reduced by SSA treatment (Figure 4C). We further used specific siRNA to knock down xCT expression and found that the knockdown of xCT could decrease oligomycin-induced cisplatin resistance (Figures 4D and 4E). Moreover, we used BSO to inhibit the biosynthesis of GSH, and found that BSO could reduce oligomycin-induced cisplatin resistance (Figure 4F). These results suggested that increased xCT expression contributed to mitochondrial dysfunction-enhanced cisplatin resistance.

### Activation of the eIF2α-ATF4 pathway contributes to mitochondrial dysfunction-induced xCT expression and high xCT expression in cisplatin-resistant cancer cells

**Figure 5:**
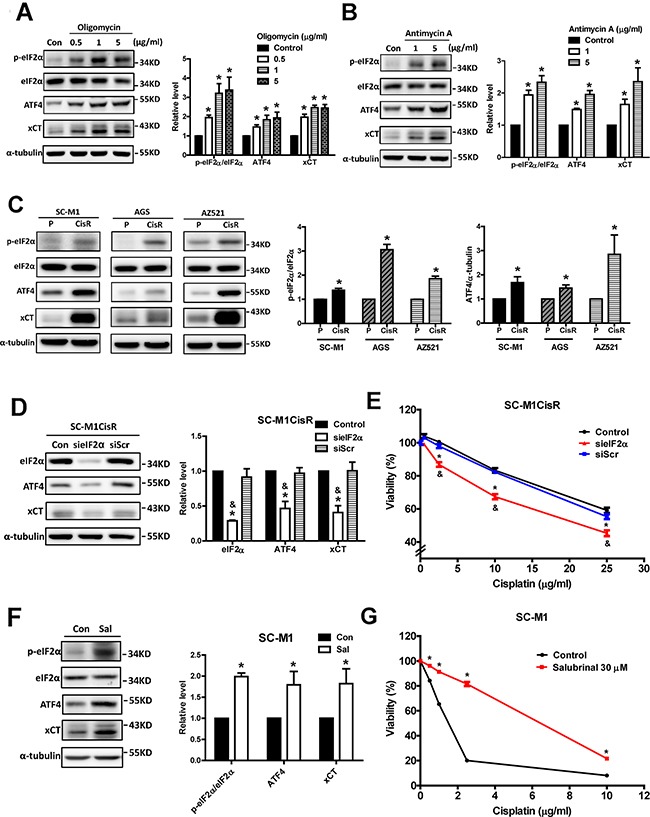
Activation of the eIF2α-ATF4 pathway contributes to mitochondrial dysfunction-induced xCT expression and high xCT expression in cisplatin-resistant cancer cells **A, B.** Western blot analysis of the eIF2α-ATF4-xCT pathway in SC-M1 cells under oligomycin (A) and antimycin A (B) treatments for 24 h, respectively. **C.** Western blot analysis of the eIF2α-ATF4-xCT pathway between parental and the cisplatin-resistant gastric cancer cells. The immunoblot values were normalized to α-tubulin. The eIF2α-ATF4-xCT pathway was determined by Western blot with specific antibodies against phosphorylated eIF2α, eIF2α, ATF4, and xCT. **D.** The specific eIF2α siRNA (60 pmol for 4 × 10^5^ cells in a 6-cm dish) was used to knock down eIF2α in the SC-M1CisR gastric cancer cells, and the eIF2α-ATF4-xCT pathway was analyzed by Western blot analysis. **E.** The eIF2α-silenced SC-M1CisR cells (sieIF2α) and the control SC-M1CisR cells were treated with cisplatin for 48 h. The cell viability was determined by SRB assay. **F.** The SC-M1 cells were treated with salubrinal (Sal, 30 μM) for 24 h and, the eIF2α-ATF4-xCT pathway was analyzed by Western blot analysis. The immunoblot values were normalized to α-tubulin. **G.** The SC-M1 cells were pre-treated with 30 μM salubrinal for 24 h, followed by co-treatment with cisplatin for 48 h. The cell viability was determined by MTT assay. Data represent the mean ± SEM of three independent experiments. *p < 0.05, compared to the control group or parental cells; & *p* < 0.05, compared to the individual siScr group.

Because it was reported that the eIF2α-ATF4 pathway increases xCT expression in response to stress conditions [16], we evaluated whether this pathway was involved in mitochondrial dysfunction-induced xCT expression. Immunoblot results showed that both the levels of phosphorylated eIF2α and ATF4 protein expression were increased by oligomycin (Figure 5A) and antimycin A (Figure 5B), respectively. These results indicated that the eIF2α-ATF4 pathway was activated by mitochondrial dysfunction. We also found that the cisplatin-resistant cells had increased eIF2α phosphorylation and ATF4 protein expression (Figure 5C). In addition, the knockdown of eIF2α by siRNA (sieIF2α) could reduce both ATF4 and xCT protein expression (Figure 5D) and could sensitize cisplatin-resistant cells to cisplatin (Figure 5E).

To elucidate whether the activation of the eIF2α-ATF4 pathway per se was sufficient for the increases in xCT expression and cisplatin resistance, we treated the cancer cells with salubrinal (an eIF2α phosphatase inhibitor) [23] to elevate the activity of this pathway. Salubrinal treatments not only increased eIF2α phosphorylation and ATF4 protein expression, but also elevated xCT expression (Figure 5F) and cisplatin resistance (Figure 5G).

**Figure 6:**
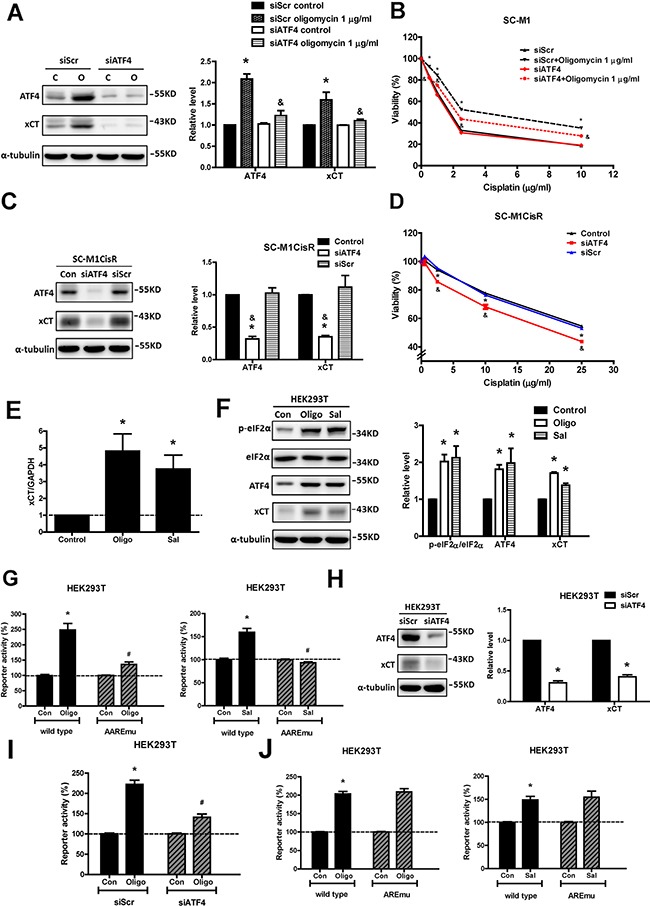
ATF4, an eIF2α downstream factor, is responsible for mitochondrial dysfunction-induced xCT expression and cisplatin resistance **A.** Western blot analysis of the expressions of ATF4 and xCT in the ATF4-silenced SC-M1 cells (siATF4) and the control cells (siRNA for non-target sequence, siScramble, siScr) under oligomycin treatment for 24 h. The immunoblot values were normalized to α-tubulin. **B.** The specific siRNA (100 pmol for 4 × 10^5^ cells in a 6-cm dish) against ATF4 was used to knock down ATF4 in the SC-M1 cells. The cells were treated with oligomycin and cisplatin for 48 h. The cell viability was determined by SRB assay. **C.** The ATF4 siRNA (100 pmol for 4 × 10^5^ cells in a 6-cm dish) was used to knock down ATF4 in the SC-M1CisR cells and the expressions of ATF4 and xCT were analyzed by Western blot analysis. **D.** The ATF4-silenced SC-M1CisR cells (siATF4) and the control SC-M1CisR cells were treated with cisplatin for 48 h. The cell viability was determined by SRB assay. **E.** qRT-PCR analysis of xCT mRNA in the HEK293T cells under 1 μg/ml oligomycin and 30 μM salubrinal treatments for 24 h. qRT-PCR values were normalized to GAPDH mRNA. **F.** Western blot analysis of the eIF2α-ATF4-xCT pathway in the HEK293T cells under 1 μg/ml oligomycin and 30 μM salubrinal treatments for 24 h. **G, J** The wild-type xCT promoter luciferase reporter (G, J), AARE-mutant (AAREmu) xCT promoter luciferase reporter (G) and ARE-mutant (AREmu) xCT promoter luciferase reporter (J) constructs were transfected using Turbofect transfection reagent. After transfection, cells were incubated with 1 μg/ml oligomycin and 30 μM salubrinal for 24 h. The reporter activity was normalized with EGFP. **H.** Western blot analysis of the expression levels of ATF4 and xCT in the ATF4-silenced HEK293T cells (siATF4). **I.** The specific siRNA (20 pmol for 4 × 10^5^ cells in 6-cm dish) against ATF4 was used to knock down ATF4 in the HEK293T cells. After ATF4 knockdown, cells were seeded at a density of 2 × 10^5^ cells per well in 6-well plates and further transfected the wild-type xCT promoter luciferase reporter construct. After transfection, cells were incubated with 1 μg/ml oligomycin for 24 h. Data represent the mean ± SEM of three independent experiments. *p < 0.05, compared to the control group or parental cells; *& p* < 0.05, compared to the individual siScr group; #*p<0.05*, compared to the oligomycin- or salubrinal-treated group.

To validate further the role of ATF4 in xCT expression, we used siRNA to knockdown ATF4 in the parental and cisplatin-resistant SC-M1 cells. We found that ATF4 knockdown could decrease the extent of oligomycin-induced xCT expression (Figure 6A), and could reduce oligomycin-induced cisplatin resistance (Figure 6B). Consistently, the knockdown of ATF4 in the cisplatin-resistant cells could decrease xCT expression (Figure 6C) and could partly reduce cisplatin resistance (Figure 6D). Furthermore, we constructed wild-type, AARE-mutant, and ARE-mutant xCT promoter luciferase reporter plasmids to demonstrate the importance of the AAREs of the xCT promoter to the mechanism. We first assessed whether oligomycin and salubrinal increase the expression levels of xCT mRNA and protein and activate the eIF2α-ATF4-xCT pathway in HEK293T cells (Figures 6E and 6F). Importantly, we found that oligomycin and salubrinal could increase the wild-type xCT promoter reporter activity but could not significantly increase the AARE-mutant xCT promoter reporter activity (Figure 6G). Moreover, ATF4 knockdown could reduce the extent of oligomycin-induced xCT promoter reporter activity (Figures 6H and 6I). These results suggested that ATF4 was involved in the mechanism. In contrast, oligomycin and salubrinal could increase the ARE-mutant xCT promoter reporter activity (Figure 6J). In addition, the protein expression of Nrf2 was not significantly increased by oligomycin treatment or in the cisplatin-resistant SC-M1 cells (Supplementary Figures S1F and S1G). These results suggested that Nrf2 was not involved in xCT expression in our system. Therefore, these results suggested that the activation of the eIF2α-ATF4 pathway could increase xCT expression and contribute to mitochondrial dysfunction-enhanced cisplatin resistance.

### GCN2, but not PERK, participates in the eIF2α-ATF4-xCT pathway, in cisplatin resistance in response to mitochondrial dysfunction and in cisplatin-resistant cells

**Figure 7:**
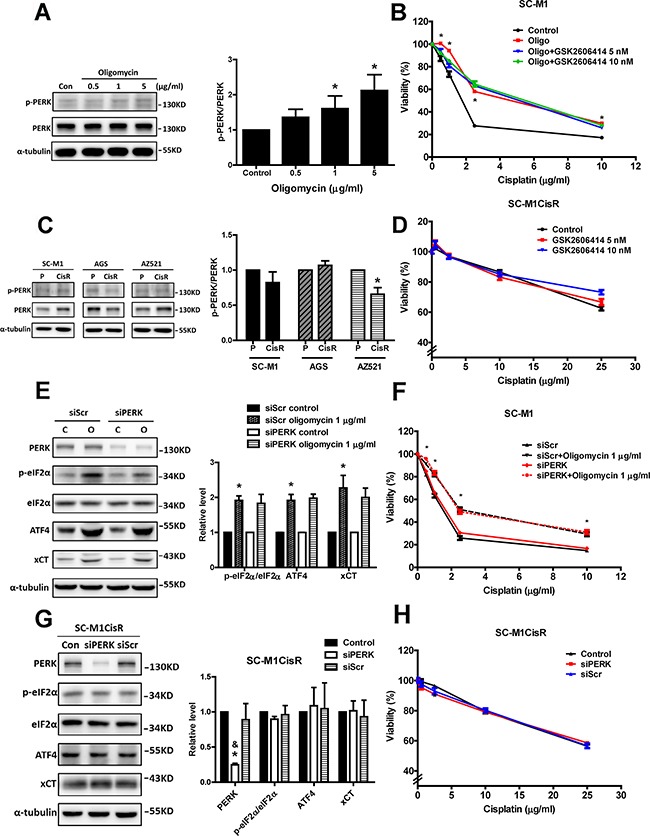
PERK does not participate in the eIF2α-ATF4-xCT pathway or cisplatin resistance in response to mitochondrial dysfunction or in cisplatin-resistant cells **A.** Western blot analysis of PERK activation in the SC-M1 cells under oligomycin treatment for 24 h. The activation of PERK was determined by Western blot with specific antibodies against phosphorylated-PERK (p-PERK) and PERK. The immunoblot values were normalized to α-tubulin. **B.** The SC-M1 cells were treated with oligomycin (Oligo, oligomycin 1 μg/ml), cisplatin, and GSK2606414 (PERK inhibitor) for 48h. The cell viability was determined by SRB assay. **C.** Western blot analysis of PERK activation between the parental and the cisplatin-resistant gastric cancer cells. The immunoblot values were normalized to α-tubulin. **D.** The SC-M1CisR cells were treated with GSK2606414 and cisplatin for 48h. The cell viability was determined by SRB assay. **E.** Western blot analysis of the eIF2α-ATF4-xCT pathway in the PERK-silenced SC-M1 cells (siPERK) and the control cells (siRNA for non-target sequence, siScramble, siScr) under oligomycin treatments for 24 h. The immunoblot values were normalized to α-tubulin. **F.** The specific siRNA (100 pmol for 4 × 10^5^ cells in 6-cm dish) against PERK was used to knock down PERK in the SC-M1 cells. The cells treated with oligomycin and cisplatin for 48 h. The cell viability was determined by SRB assay. **G.** The PERK siRNA (100 pmol for 4 × 10^5^ cells in a 6-cm dish) was used to knock down PERK in the SC-M1CisR cells, and the eIF2α-ATF4-xCT pathway was analyzed by Western blot analysis. **H.** The PERK-silenced SC-M1CisR cells (siPERK) and the control SC-M1CisR cells were treated with cisplatin for 48 h. The cell viability was determined by SRB assay. Data represent the mean ± SEM of three independent experiments. *p < 0.05, compared to the control group or parental cells; *& p < 0.05*, compared to the individual siScr group.

We further explored whether the eIF2α kinase PERK was involved in mitochondrial dysfunction-enhanced cisplatin resistance. We found that oligomycin treatments slightly increased PERK phosphorylation (Figure 7A). However, a specific PERK inhibitor (GSK2606414) did not significantly reduce oligomycin-induced cisplatin resistance (Figure 7B). Moreover, the PERK phosphorylation levels were not significantly increased in the cisplatin-resistant cancer cells (Figure 7C). The change in PERK phosphorylation in cisplatin-resistant AZ521 cells might result from the variation of total PERK protein level. The detailed reason remains unclear. In addition, GSK2606414 treatment did not significantly sensitize the cisplatin-resistant cancer cells to cisplatin (Figure 7D). We further used the genetic knockdown of PERK to confirm the role of PERK in mitochondrial dysfunction-enhanced cisplatin resistance. The results revealed that PERK knockdown did not decrease the oligomycin-induced activation of the eIF2α-ATF4-xCT pathway (Figure 7E) and did not reduce oligomycin-induced cisplatin resistance (Figure 7F). In the cisplatin-resistant cells, PERK knockdown could not decrease the phosphorylation of eIF2α or the expression levels of ATF4 and xCT (Figure 7G), and it could not reduce cisplatin resistance (Figure 7H). These results suggested that PERK might not play a major role in oligomycin-induced cisplatin resistance.

**Figure 8:**
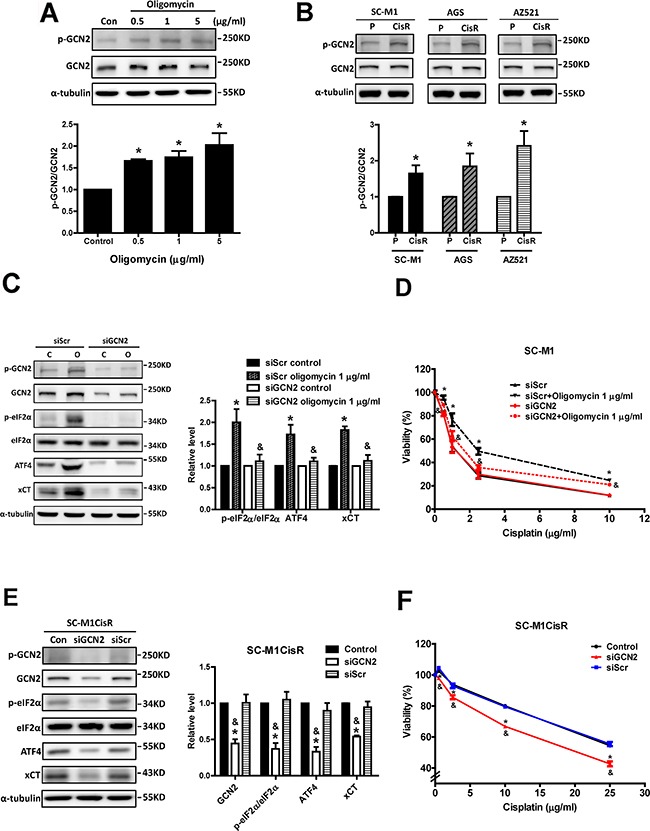
GCN2 participates in the eIF2α-ATF4-xCT pathway and cisplatin resistance in response to mitochondrial dysfunction and in cisplatin-resistant cells **A.** Western blot analysis of GCN2 activation in SC-M1 cells under oligomycin treatments for 24 h. **B.** Western blot analysis of GCN2 activation between the parental and the cisplatin-resistant gastric cancer cells. The activation of GCN2 was determined by Western blot with specific antibodies against phosphorylated GCN2 (p-GCN2) and GCN2. The immunoblot values were normalized to α-tubulin. **C.** Western blot analysis of the GCN2-eIF2α-ATF4-xCT pathway in the GCN2-silenced SC-M1 cells (siGCN2) and the control cells (siRNA for non-target sequence, siScramble, siScr) under oligomycin treatments for 24 h. The immunoblot values were normalized to α-tubulin. **D.** The specific siRNA (60 pmol for 4 × 10^5^ cells in a 6-cm dish) against GCN2 was used to knock down GCN2 in the SC-M1 cells. The cells were treated with oligomycin and cisplatin for 48 h. The cell viability was determined by SRB assay. **E.** The GCN2 siRNA (60 pmol for 4 × 10^5^ cells in a 6-cm dish) was used to knock down GCN2 in the SC-M1CisR cells, and the GCN2-eIF2α-ATF4-xCT pathway was analyzed by Western blot analysis. **F.** The GCN2-silenced SC-M1CisR cells (siGCN2) and the control SC-M1CisR cells were treated with cisplatin for 48 h. The cell viability was determined by SRB assay. Data represent the mean ± SEM of three independent experiments. *p < 0.05, compared to the control group or parental cells; *& p* < 0.05, compared to the individual siScr group.

In contrast, we found that oligomycin treatments could increase GCN2 phosphorylation and the phosphorylated GCN2 level was higher in the cisplatin-resistant cells than in the parental cells (Figures 8A and 8B). In addition, the knockdown of GCN2 decreased the oligomycin-induced activation of the eIF2α-ATF4-xCT pathway (Figure 8C) and reduced oligomycin-induced cisplatin resistance (Figure 8D). These results suggested that GCN2 was involved in the mitochondrial dysfunction-induced eIF2α-ATF4-xCT pathway and in cisplatin resistance. Consistently, the knockdown of GCN2 in the cisplatin-resistant cells could decrease the phosphorylation of eIF2α and the expression levels of ATF4 and xCT (Figure 8E) and could partly reduce cisplatin resistance (Figure 8F). These results suggested that GCN2 was involved in the activation of the eIF2α-ATF4-xCT pathway and cisplatin resistance in human gastric cancer cells.

### ROS mediates the mitochondrial dysfunction-induced activation of the GCN2-eIF2α-ATF4-xCT pathway

**Figure 9:**
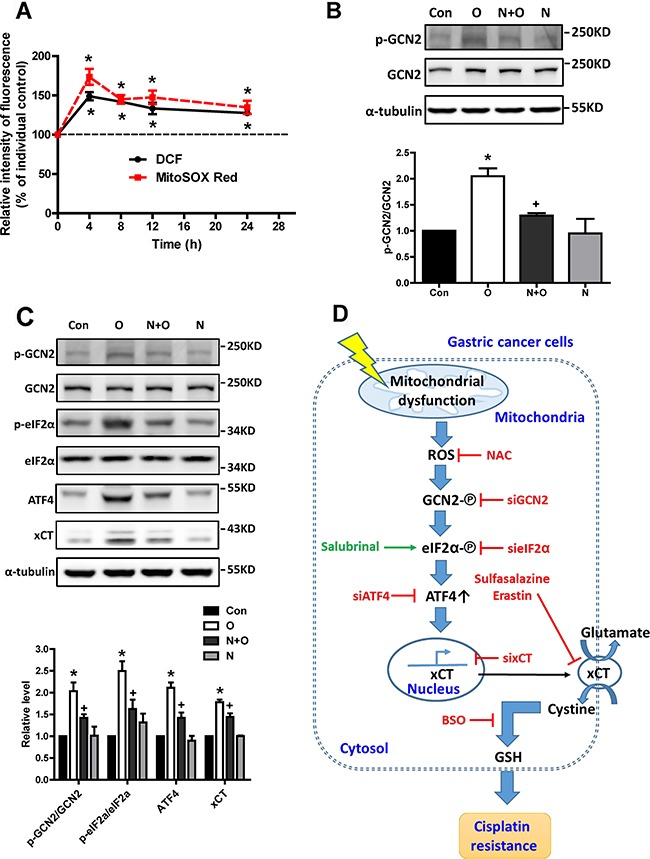
ROS mediates mitochondrial dysfunction-induced activation of the GCN2-eIF2α-ATF4-xCT pathway **A.** The SC-M1 cells were treated with oligomycin for 4-24 h. After incubation for the indicated durations, the cells were stain with DCFH-dA 5 μM for 30 min or MitoSOX Red 10 μM for 10 min and were further collected and analyzed by flow cytometry. **B.** Western blot analysis of GCN2 activation in the SC-M1 cells under oligomycin (O, 1 μg/ml) and N-acetyl cysteine (NAC, N, 5 mM) treatments for 4 h. **C.** Western blot analysis of the GCN2-eIF2α-ATF4-xCT pathway in the SC-M1 cells under oligomycin (O, 1 μg/ml) and N-acetyl cysteine (NAC, N, 5 mM) treatments for 24 h. The immunoblot values were normalized to α-tubulin. **D.** The scheme of the ROS-activated GCN2-eIF2α-ATF4-xCT pathway was involved in mitochondrial dysfunction-induced mitochondrial retrograde signaling and further conferred cisplatin resistance in human gastric cancer cells. Data represent the mean ± SEM of three independent experiments. *p < 0.05, compared to the control group; *+ p* < 0.05, compared to the oligomycin group.

Mitochondrial electron transport chains constitute the major intracellular ROS source and increased ROS levels are usually obtained in cells with mtDNA mutations and mitochondrial dysfunction [4]. Moreover, mitochondrial dysfunction due to oligomycin could increase intracellular (DCF) and mitochondrial (MitoSOX Red) ROS (Figure 9A). Hence, we further determined whether ROS participated in mitochondrial dysfunction-induced GCN2 activation. We found that the antioxidant NAC could reverse the phosphorylation of GCN2 by oligomycin treatment (Figure 9B). Furthermore, we found that NAC could reverse the activation of the GCN2-ATF4- eIF2α-xCT pathway by oligomycin (Figure 9C). These results indicated that ROS mediated the mitochondrial dysfunction-induced activation of the GCN2-ATF4- eIF2α-xCT pathway.

## DISCUSSION

In this study, we demonstrated that mitochondrial dysfunction induced by oligomycin and antimycin A might enhance cisplatin resistance in human gastric cancer cells through increased xCT expression and intracellular GSH levels, as well as through the ROS activation of the GCN2-eIF2α-ATF4 pathway. Moreover, high xCT expression and the activation of the GCN2-eIF2α-ATF4 pathway were observed in cisplatin-resistant gastric cancer cells. In addition, high xCT expression in gastric cancer was associated with the poor prognosis of patients under adjuvant chemotherapy treatment. These results provided evidence for the first time to suggest that the ROS-activated GCN2-eIF2α-ATF4-xCT pathway is retrograde signaling that contributes to mitochondrial dysfunction-enhanced cisplatin resistance in human gastric cancer cells (Figure 9D). In our previous studies, we found that mitochondrial DNA mutations and defects in mitochondrial enzymes in gastric cancers might contribute to cancer progression and chemoresistance [5, 6]. In this study, we further demonstrated that xCT might be an important link between mitochondrial dysfunction and cisplatin resistance.

The outcome of the integrated stress response induced by different mitochondrial stressors can be protective or deleterious, likely depending on the intensity and type of the mitochondrial insult or the cell type [7]. Irreversible and severe cellular damage might induce cell death. In the present study, we demonstrated that mitochondrial dysfunction induced by low concentrations of oligomycin and antimycin A could increase xCT expression by activating the eIF2α-ATF4 pathway, resulting in cisplatin resistance in three different gastric cancer cell lines. Our results suggested that mild mitochondrial dysfunction could activate the protective response in human gastric cancer cells. The findings were consistent with the previous report that heteroplasmic, but not homoplasmic, mtDNA mutation could promote tumor cell growth [24]. The severe mitochondrial defects caused by homoplasmic mtDNA mutations might be deleterious to cancer development.

It has been demonstrated that the eIF2α-ATF4 pathway is essential for tumor cell survival and proliferation in response to various stress conditions [18, 25]. In addition, the increased expression of ATF4 might promote the expression of the other stress-response genes, such as CEBP homologous (CHOP), growth arrest and DNA damage-inducible protein 34 (GADD34), immunoglobulin heavy chain-binding protein (BiP), and other transcription factors [26, 27]. Therefore, it is possible that these stress-response proteins could provide the mechanisms by which cancer cells exhibit resistance to a board range of chemotherapies.

PERK and GCN2 were reported to be activated by different mitochondrial stressors [19, 28]. In the present study, we found that GCN2, but not PERK, majorly involves in the mitochondrial dysfunction-activated eIF2α-ATF4-xCT pathway and cisplatin resistance in gastric cancer cells. GCN2 is a high molecular weight protein and can be activated by uncharged tRNA or impaired expression of mtDNA in different mammalian cell types [29, 30]. Moreover, hydrogen peroxide can increase GCN-2 activity through a mechanism for the tRNA synthetase domain in the *C. elegans* and yeasts [31-33]. Under stress conditions, GCN2 could be auto-phosphorylated and could exhibit its kinase activity [34], which phosphorylated eIF2α [18]. In the present study, we demonstrated that mitochondrial dysfunction-induced ROS could activate GCN2 in human gastric cancer cells.

The system x_c_^−^ cystine/glutamate antiporter is responsible for the cystine uptake from extracellular environment to maintain thiol-containing molecule homeostasis, particularly of GSH [13]. The imported cystine is reduced to cysteine, which is essential for GSH biosynthesis. In the present study, two inhibitors of the system x_c_^−^ antiporter (sulfasalazine and erastin) and an inhibitor of GSH biosynthesis (BSO) can reduce mitochondrial dysfunction-enhanced cisplatin resistance, suggesting that elevated intracellular GSH levels due to the system x_c_^−^ antiporter might contribute to chemoresistance. The GSH-GSSG system not only provided an important redox buffer to scavenge cisplatin-induced oxidative stress [35], but it also deactivated cisplatin [11]. Moreover, the increased GSH might increase DNA repair and protect cancer cells from cisplatin-induced cytotoxicity [12]. Therefore, our findings provided a link between mitochondrial dysfunction and cancer progression by enhancing chemoresistance.

Increased xCT expression has been found to be essential for cancer proliferation and malignant progression in certain cancer cells [36, 37]. Inhibiting xCT attenuated stem-like cell behavior and metastatic progression in breast cancer [38]. Immunotargeting xCT increased the chemosensitivity of radioresistant cancer stem cells to doxorubicin *in vivo*. The expression of xCT at the plasma membrane of cancer cells was found to be stabilized by CD44v isoform, resulting in increased GSH synthesis [39]. Moreover, CD44v was implicated in metaplasia-carcinoma sequence progression in the stomach and conferred resistance to various types of cellular stress [40]. CD44 genetic knockdown or xCT inhibitors could suppress the development of metaplasia and subsequent gastric tumor growth. During gastric cancer development, chronic gastric inflammation and the histopathologic progression of stomach epithelium cells might constitute critical risk factors for the development of metaplasia and eventually gastric cancer. Therefore, the expression of xCT and CD44v might serve as biomarkers for gastric cancer progression.

In the evolution of systemic chemotherapy for gastric cancer, cisplatin-containing combinations were eventually shown to be superior to non-cisplatin-containing regimens, and they have become the reference regimens for advanced gastric cancer [41]. Combination chemotherapy has better response rates, but the time to progression is still only approximately 3-6 months [41]. In general, clinical trials assessing the efficacy of a variety of 2^nd^-line chemotherapy regimens after the failure of the 1^st^ -line regimens have shown that the response rates are lower than those of patients without previous treatment, and the toxicity rates tend to be higher [42, 43]. Hence, chemotherapy resistance remains an obstacle to subsequent treatment despite chemotherapy for gastric cancer having acceptable clinical efficacy. xCT not only has been proposed as a possible target for cisplatin resistance but also for comprehensive chemoresistance or targeted therapy resistance [39, 44, 45]. Furthermore, another remaining barrier for chemotherapy is the selectivity for killing cancer cells based on cancer-specific features [46]. Cancer stem cells might enhance ROS scavenger capacity through increased xCT expression and GSH synthesis. Inhibiting xCT can attenuate the stemness properties and metastatic progression in various cancers [38, 39]. Therefore, these findings suggested that xCT might be a poor prognostic factor for gastric cancer patients undergoing adjuvant chemotherapy treatment, and xCT might be a potential target for chemoresistance.

In conclusion, we identified for the first time that the ROS-activated GCN2-eIF2α-ATF4-xCT pathway contributes to mitochondrial dysfunction-enhanced cisplatin resistance in human gastric cancer cells. The GCN2-eIF2α-ATF4-xCT pathway might be a potential drug target for reducing chemoresistance and improving gastric cancer therapy.

## MATERIALS AND METHODS

### Chemicals and reagents

Oligomycin, sulfasalazine (SSA, an xCT inhibitor [36]), erastin (an xCT inhibitor [47]), N-acetyl cysteine (NAC), L-amino acid kit, 5-flurouracil (5-FU), antimycin A, sulforhodamine B (SRB), buthionine sulphoximine (BSO), 3-(4,5-cimethylthiazol-2-yl)-2,5-diphenyl tetrazolium bromide (MTT), acetic acid, trichloroacetic acid (TCA), aprotinin, phenylmethanesulfonyl fluoride (PMSF), sodium orthovanadate (Na_3_VO_4_), dimethyl sulfoxide (DMSO), Triton X-100, Bradford reagent, bovine serum albumin (BSA), and α-tubulin antibody were purchased from Sigma-Aldrich (St. Louis, MO, USA). Sodium dodecyl sulfate (SDS), Tris-HCl buffer, and sodium chloride (NaCl) were purchased from Merck Millipore (Billerica, MA, USA). Cisplatin was obtained from Fresenius Kabi Oncology (Distt. Solan, H.P., India). GSK2606414 (a PERK inhibitor [48]) was purchased from Calbiochem™, Merck Millipore (Billerica, MA, USA). ON-TARGET plus™ SMARTpool EIF2S1 (eIF2α), ATF4, SLC7A11 (xCT), EIF2AK3 (PERK), EIF2AK4 (GCN2) and non-target (scramble) siRNAs were purchased from GE Healthcare Dharmacon (Lafayette, CO, USA). 2′7′-dichlorodihydrofluorescein diacetate (DCFH-dA) and MitoSOX Red were purchased from Molecular Probe™, Invitrogen™, Thermo Fisher Scientific (Eugene, Oregon, USA). Specific primers for real-time polymerase chain reaction (PCR) were purchased from Mission Biotech (Taipei, Taiwan). Salubrinal (an eIF2α phosphatase inhibitor [23]) and the p-PERK Thr981 antibody were purchased from Santa Cruz Biotechnology (Santa Cruz, CA, USA). Antibodies against GCN2 and PERK were purchased from Cell Signaling Technology (Beverly, MA, USA). Antibodies against p-GCN2 (Thr899) and xCT were purchased from Abcam (Cambridge, MA, USA). ATF4 antibody was purchased from Proteintech Group (Rosemont, IL, USA). Antibodies against p-eIF2α (Ser52) and eIF2α were purchased from Invitrogen™, Thermo Fisher Scientific (Camarillo, CA, USA).

### Cell culture and establishment of resistant gastric cancer cell lines

The human gastric cancer cell lines SC-M1, AGS, and AZ521 were cultured in RPMI 1640 medium with 10 % fetal bovine serum (FBS), and 1 % penicillin/streptomycin (P/S) and were incubated in a humidified 37 °C incubator with 5 % CO_2_. Human embryonic kidney 293T (HEK293T) cells were cultured in Dulbecco's modified Eagle's medium (DMEM) supplemented with 10 % FBS, 2 mmol/l L-glutamine, 10 mmol/l non-essential amino acids (NEAAs), and 1 % penicillin/streptomycin (P/S) at 37 °C in a humidified 5 % CO_2_ incubator. RPMI1640 and DMEM were purchased from Gibco™, Thermo Fisher Scientific (Grand Island, NY, USA). FBS, P/S, L-glutamine, and NEAA were purchased from Biological Industries (Kibbutz Beit Haemek, Israel). Cystine depletion experiments were performed by manually compounding individual cultured medium. Cystine depletion medium was formulated with or w/o amino acid RPMI 1640 (United States Biological, Swampscott, MA, USA) and the subsequent addition of the same amount of amino acid (except for cystine), according to the formula for original RPMI1640. To establish cisplatin-resistant (CisR) gastric cancer cells, the parental gastric cancer cells were treated with slowly increasing concentrations of cisplatin until the cells could tolerate the IC50 of cisplatin for 6 months. After attaining the IC50 stage, the cisplatin-resistant gastric cancer cells were cultured in medium containing the IC20 concentration of cisplatin medium for subsequent experiments (SC-M1CiSR: 0.5 μg/ml; AGSCisR: 1 μg/ml; AZ521CisR: 1 μg/ml). Before the function assay and drug sensitivity testing, cells were cultured in cisplatin-free medium for 3 days. The cisplatin resistance of drug-resistant gastric cancer cells was routinely examined for the determination of the sensitivity to cisplatin by SRB assay every 2 months.

### Determination of cell viability

Cell viability was determined by SRB or MTT assay. Cells were seeded in 96-well cell culture plates (Corning Inc., Corning, NY, USA) at a density of 3000-5000 cells per well and were cultured 24 hours prior to drug treatment. In the SRB assay, cells were fixed with 10 % TCA after the indicated period of treatment. After washing with distilled water, cells were stained with 0.057 % SRB and were washed with 1 % acetic acid. The cell viability was assessed by OD determination at 510 nm using a microplate reader. In the MTT assay, after the indicated period, the medium was discarded and replaced with an equal volume (200μl) of fresh medium containing MTT and was incubated at 37 °C for 1 h in the dark. Next, the MTT medium was discarded, and DMSO was added to dissolve the formazan that was produced. The cell viability was determined by a colorimetric method using a microplate reader at the absorption wavelength of 570 nm.

### Determination of intracellular glutathione (GSH)

Cells were seeded in a 10-cm dish (Corning Inc., Corning, NY, USA) at a density of 1 × 10^6^ cells and were cultured 24 h prior to drug treatment. The sample was deproteinized with 5 % 5-sulfosalicylic acid solution. The cellular level of glutathione was determined using a Glutathione Assay Kit (Sigma-Aldrich, St. Louis, MO, USA), according to the manufacturer's protocol.

### Real-time reverse transcription (RT)-polymerase chain reaction (q-RT PCR)

Cellular total RNA was extracted from cells using TRIzol reagent, following the manufacturer's instructions (Invitrogen™, Thermo Fisher Scientific, Carlsbad, CA, USA). Total RNA (20 μg) was used in the RT reaction by RevertAid™ reverse transcriptase (Thermo Fischer Scientific, Waltham, MA, USA). The real-time PCR amplifications were performed in the StepOne™ System (Applied Biosystems™ real-time PCR Instrument, Thermo Fisher Scientific) using KAPA SYBR FAST qPCR Kits (Kapa Biosystems, Wilmington, MA, USA). The primer sequences were SLC7A11 (xCT), forward: TCATTGGAGCAGGAATCTTCA; reverse: TTCAGCATAAGAGAAAGCTCCA; and glyceraldehyde 3-phosphate dehydrogenase (GAPDH), forward: CCGTCTAGAAAAACCTGCC; reverse: GCCAAATTCGTTGTCATACC. The reaction mixture was first denatured at 95 °C for 3 min. The PCR condition was 95 °C for 3 s, and 60 °C for 30 s each cycle (40 cycles). Gene expression levels were calculated by the 2^−ΔΔCt^ method and were normalized to the level of GADPH in each sample.

### Western blot analysis

Cell lysate was extracted using radioimmunoprecipitation assay buffer (RIPA buffer: 50 mM Tris-HCl buffer, pH 7.5, containing 0.15 M NaCl, 0.5 % sodium deoxycholate, 0.5 % SDS, 0.1% Triton X-100, 10 μg/ml aprotinin, 2 mM ethylenediaminetetraacetic acid [EDTA], 2 mM Na_3_VO_4_, and 1 mM PMSF). The concentration of protein was determined using Bradford reagent with BSA as the standard. Twenty micrograms of lysate protein were resolved by 8-12 % SDS-PAGE, transferred onto a polyvinylidene difluoride membrane (PVDF membrane, Biotrace™, PALL Life sciences, Ann Arbor, MI, USA), and immunoblotted with antibodies. The protein contents were visualized using a chemiluminescence kit (Immobilon Western Chemiluminescence HRP Substrates, Merck-Millipore, Billerica, MA, USA). The images of Western blot were observed using a Luminescence/Fluorescence Imaging System (GE Healthcare), and the signal intensities were quantified using Multi Gauge image analysis software (Fujifilm).

### Small interfering (siRNA)-mediated specific gene knockdown

Cells were seeded in 6-cm dish (Corning Inc., Corning, NY, USA) at a density of 4 × 10^5^ cells and were cultured overnight in antibiotic-free complete medium. Lipofectamine RNAi MAX reagent (Invitrogen™, Thermo Fisher Scientific, Carlsbad, CA, USA) and siRNA (60 pmol) were diluted in OPTI-MEM medium (Gibco™, Thermo Fisher Scientific, Grand Island, NY, USA). First, the diluted siRNA was mixed with Lipofectamine for 10 min at room temperature. Then, the siRNA-lipid complex was added to the medium and was incubated for 48 h for subsequent experiments. The specific ON-TARGET plus™ SMARTpool EIF2S1 (eIF2α, L-015389), ATF4 (L-005125), SLC7A11 (xCT, L-007612), EIF2AK3 (PERK, L-004883), EIF2AK4 (GCN2, L-005314) and non-target (scramble, D-001810) siRNAs were used in these experiments.

### Kaplan-Meier plotter analysis

This online database was based on the GEO (Affymetrix microarrays only), EGA and TCGA databases. The two patient cohorts were compared by Kaplan-Meier (KM) survival plots, and hazard ratios with 95 % confidence intervals and log rank *p* values were calculated using online software, as a previous study described [49]. In the gastric cancer database, the specific gene, xCT (SLC7A11, gene symbol Affy ID 209921_at), was used in this KM analysis. In the specific clinical cohorts receiving 5-FU based adjuvant therapy, a total of 153 patients were analyzed for overall survival (OS) and progression free survival (PFS) by KM analysis.

### Detection of the level of intracellular ROS and mitochondrial ROS

DCFH-dA and MitoSOX Red were used to determine the intracellular and mitochondrial ROS, respectively. After incubation with 5 μM DCFH-dA for 30 min or 10 μM MitoSOX Red for 10 min, cells were washed with PBS, trypsinized, and re-suspended in PBS, as in previous methods. The DCF fluorescence intensity at FL1 and the MitoSOX Red fluorescence intensity at FL2 were determined by flow cytometry. A FACS Calibur flow cytometer (Becton Dickinson Bedford, MA, USA) equipped with a 488-nm argon laser was used for the flow cytometric analysis. The excitation wavelength was set at 488 nm. In each measurement, a minimum of 15000 cells were analyzed. Data were acquired and analyzed using Cell Quest software (Becton Dickinson). The relative change in the mean fluorescence intensity was calculated as the ratio between the mean fluorescence intensity in the channel of the treated cells and that in the channel of the control cells.

### Human xCT promoter construction and reporter luciferase activity assay

Cells (2 × 10^5^) were seeded in 6-well culture plates and were transfected using Turbofect transfection reagent (Thermo Fisher Scientific), according to the manufacturer's instructions. Cells were co-transfected with 4 μg of xCT reporter vector and 4 μg of pEGFP-C2 per well. The wild-type, ARE-mutant, and AARE-mutant xCT promoter constructs in the pGL3 luciferase reporter vector (Promega, Madison, WI, USA) are described in Supplementary Figure S1H. The human xCT promoters with ARE mutants or AARE mutants were created by site-direct mutagenesis, according to previous studies [15, 50, 51]. After transfection, cells were incubated in medium containing the indicated drugs or vehicle for 24 h and were collected for the determination of luciferase activity. The reporter assay was performed with the Promega luciferase assay system (Madison, WI, USA), following the manufacturer's manual. The green fluorescence intensity of pEGFP was used to normalize for transfection efficiency, and the relative ratio of luciferase to pEGFP was indicated as the reporter activity.

### Statistical analysis

All of the data are presented as the mean ± SEM. Sigmaplot software, version 10.0 (Systat Software), and GraphPad PRISM software, version 5 (GraphPad Software), were used for all statistical analyses. The statistical significance of the differences between two groups was evaluated using Student's t test. A *p* value <0.05 was considered to be statistically significant.

## SUPPLEMENTARY MATERIALS FIGURE



## Abbreviations

5-FU: 5-fluorouracil
ARE: antioxidant response element
AARE: amino acid response element
ATF4: activating transcription factor 4
ATP: adenosine triphosphate
BiP: immunoglobulin heavy chain-binding protein
BSA: bovine serum albumin
BSO: buthionine sulphoximine
CHOP: CEBP homologous
CisR: cisplatin-resistant
DCFH-dA: 2′7′-dichlorodihydrofluorescein diacetate
DMEM: Dulbecco's modified Eagle's medium
DMSO: dimethyl sulfoxide
EDTA: ethylenediaminetetraacetic acid
eIF2α: eukaryotic initiation factor 2α
FBS: fetal bovine serum
GADD34: DNA damage-inducible protein 34
GADPH: glyceraldehyde 3-phosphate dehydrogenase
GCN2: general control nonderepressible-2
GSH: glutathione
HR: hazard ratio
HRI: heme-regulated inhibitor eIF2 kinase
MDR: multidrug resistance protein
MRP: multidrug resistance-associated protein
mtDNA: mitochondrial DNA
MTT: 3-(4,5-cimethylthiazol-2-yl)-2,5-diphenyl tetrazolium bromide
NAC: N-acetyl cysteine
NEAA: non-essential amino acids
Nrf2: nuclear factor erythroid 2-related factor-2
OS: overall survival
P/S: penicillin/streptomycin
PCR: polymerase chain reaction
PERK: protein kinase R-like endoplasmic reticulum kinase
PFS: progression free survival
PKR: dsRNA-activated protein kinase R
PMSF: phenylmethanesulfonyl fluoride
PVDF: polyvinylidene difluoride
ROS: reactive oxygen species
RT: reverse transcription
SDS: sodium dodecyl sulfate
SRB: sulforhodamine B
SSA: sulfasalazine
TCA: trichloroacetic acid
