# Supplementary material for: Mitochondrial dysfunction enhances cisplatin resistance in human gastric cancer cells via the ROS-activated GCN2-eIF2α-ATF4-xCT pathway
Source: Oncotarget. 2016 Sep 30;7(45):74132–51. doi: 10.18632/oncotarget.12356 (PMC5342041; doi:10.18632/oncotarget.12356)
Supplement: Supplementary file 1 [file oncotarget-07-74132-s001.pdf]

Mitochondrial dysfunction enhances cisplatin resistance in human gastric cancer cells via the ROS-activated GCN2-eIF2α-ATF4-xCT pathway

SUPPLEMENTARY MATERIALS AND METHODS

Western blot analysis

MDR antibody was used to analyze the MDR1 protein expression by Western blot analyses. MDR and Nrf2 antibodies were purchased from Santa Cruz Biotechnology (Santa Cruz, CA, USA).

MRP efflux assay

The MRP-mediated efflux activity in cultured cells was determined using 5-carboxylfluorescein diacetate (5-CFDA) as a substrate. On a 6-well plate, 0.25 x 10^6 cells per well were seeded, and sub-confluent cells were cultured 24 h prior to determination. Sub-confluent cells were incubated with 5-CFDA in medium at 37 °C in a CO_2 incubator for 30 min. After two washes with ice-cold PBS in the dark, the cells were incubated with 5-CFDA -free medium in the presence of tested compounds at 37 °C in a CO_2 incubator for 30 min. After two washes with ice-cold PBS in the dark, cells were analyzed at 485 nm for excitation and at 538 nm. The calculated formula was as follows:

5-CFDA efflux % $=[(5\text{-CF})_{0min}-(5\text{-CF})_{30min}]/(5\text{-CF})_{0min.}$

Rh123 efflux assay

Cells were seeded on a 12-well plate (1 x 10^5/well) overnight and then were incubated with 5 µM Rh123 for 1 h. After 3 washes with ice-cold phosphate- buffered saline (PBS) in the dark, cells were incubated with Rh123-free medium in the absence and presence of 100 µM verapamil (a p-glycoprotein [P-gp, MDR1] inhibitor) for 3 h. The fluorescence of Rh123 retained in the cells was measured (Ex: 485 nm; Em: 538 nm) and the percentage of Rh123 exported from the cells (Efflux) was determined as the decrease in cellular Rh123. The efflux function of P-gp was monitored in terms of the decrease of export of Rh123 in the presence of verapamil ($\text{Efflux}_{\text{absence of verapamil}} - \text{Efflux}_{\text{+verapamil}}$).

SUPPLEMENTARY FIGURES

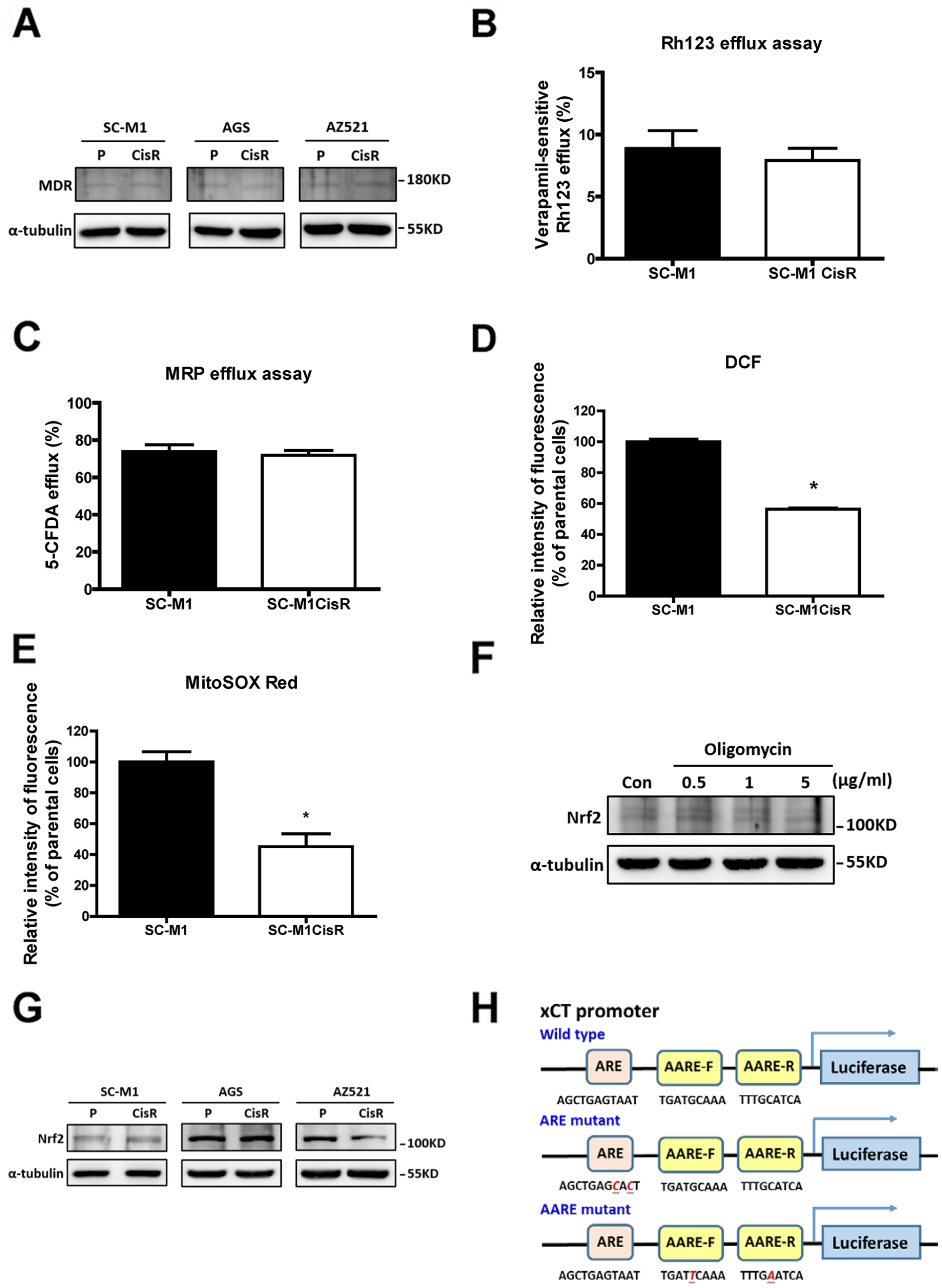

Supplementary Figure S1: A. Western blot analysis of MDR protein expression between the parental and cisplatin-resistant gastric cancer cells. The immunoblot values were normalized to α-tubulin. **B.** 1 x 10^5 cells per well were seeded in 12-well plates, and sub-confluent cells were cultured 24 h prior to determination. The efflux function of P-gp (MDR1) was monitored in terms of the decrease of the export of Rh123 in the presence of verapamil (Efflux $_{\text{absence of verapamil}}$ – Efflux$_{\text{+verapamil}}$). **C.** 0.25 x 10^6 cells per well were seeded in 6-well plates, and sub-confluent cells were cultured 24 h prior to determination. MRP efflux assay was performed by calculated 5-CFDA efflux percentage. (D, E) After being cisplatin-free for 3 days, cells were seeded at a density of 2 x 10^5 per well in 6-well plates. After culture overnight, cells were stained with DCFH-dA 5 μM for 30 min **D.** or MitoSOX Red 10 μM for 10 min **E.** and were further collected and analyzed by flow cytometry. **F.** Western blot analysis of Nrf2 protein expression in the SC-M1 cells under oligomycin treatment for 24 h. **G.** Western blot analysis of Nrf2 protein expression between the parental and cisplatin-resistant gastric cancer cells. The immunoblot values were normalized to α-tubulin. **H.** Diagram of wild-type, ARE-mutant, and AARE-mutant xCT promoter luciferase reporter constructs. Data represent the mean ± SEM. *$p < 0.05$, compared to the parental cells.
